# Traditional Approach vs Posterior Approach for Ankle Fractures Involving the Posterior Malleolus

**DOI:** 10.1177/1071100720969431

**Published:** 2020-11-17

**Authors:** Kristian Pilskog, Teresa Brnic Gote, Heid Elin Johannessen Odland, Knut Andreas Fjeldsgaard, Håvard Dale, Eivind Inderhaug, Jonas Meling Fevang

**Affiliations:** 1 Orthopedic Department, Haukeland University Hospital, Bergen, Norway; 2 Department of Physiotherapy, Haukeland University Hospital, Bergen, Norway

**Keywords:** ankle fracture, posterior malleolus, posterolateral, fixation, PROM, SEFAS, operative, outcome

## Abstract

**Background:**

In the past, posterior malleolus fragments (PMFs) commonly have been indirectly reduced and fixed when fragments involve 25% or more of the tibial articular surface, while smaller fragments were left unfixed. The posterior approach has become increasingly popular and allows fixation of even smaller fragments. This study compares clinical outcome for the 2 treatment strategies.

**Methods:**

Patients with ankle fractures involving a PMF treated from 2014 to 2016 were eligible for inclusion. Patients were allocated to group A (treated with a posterior approach) or group B (treated with the traditional approach) according to the treatment given. A one-to-one matching of patients from each group based on the size of the PMF was performed. Patient charts were reviewed, and outcome evaluation was performed clinically, radiographically, and by patient-reported outcome measures (PROMs; Self-Reported Foot and Ankle Score, RAND-36, visual analog scale [VAS] of pain, and VAS of satisfaction). Forty-three patients from each group were matched. Median follow-up was 26 (interquartile range [IQR], 19-35) months postoperatively.

**Results:**

The median PMF size was 17% (IQR, 12-24) in both groups, and they reported similar results in terms of PROMs. Fixation of the PMF was performed in 42 of 43 (98%) patients in group A and 7 of 43 (16%) patients in group B (*P* < .001). The former group more frequently got temporary external fixation (56% vs 12%, *P* < .01) and less frequently had syndesmotic fixation (14% vs 49%, *P* < .01), and they had less mechanical irritation and hardware removal but more noninfectious skin problems (28% vs 5%, *P* < .01). Median time from injury to definitive surgery (8 vs 0 days, *P* < .001) and median length of stay (12 vs 3 days, *P* < .001) were longer in group A.

**Conclusion:**

Comparison of treatment strategies for ankle fractures involving the posterior malleolus showed similar results between patients treated with a traditional approach and a posterior approach.

**Level of Evidence:**

Level III, retrospective comparative study.

Ankle fractures constitute 9% of all fractures and have an incidence of approximately 107 to 187 per 100 000 persons per year.^9,22^ A posterior malleolar fragment (PMF) is present in up to 46% of Weber B and Weber C fractures.^2^ Traditionally, the recommended cutoff for fixation of the PMF has been fragment size over 25% of the distal tibial articular surface.^31,37^ Biomechanical studies have displayed that the posterior 25% of the articular surface is not involved in weightbearing during dorsi- and plantarflexion of the ankle.^40^

Poor clinical outcomes for trimalleolar fractures have been reported in several studies.^41,46,54^ For this reason, the indication and choice of intervention for these fractures have been the object of increased interest in recent years. The PMF has traditionally been treated with closed, indirect, reduction, and, if needed, anteroposterior screw fixation.^52^ Despite lack of solid evidence, there has been a trend toward use of a posterior approach allowing open reduction and internal fixation (ORIF).^13,20,29^ It is advocated that this approach allows more anatomical reduction of the PMF and fixation of fragments smaller than 25%.^48^ In addition, fixation of the distal fibular fracture through the same incision gives good soft tissue coverage by the peroneal muscles.^51^ The posterior inferior tibiofibular ligament (PITFL) attaches to the posterior malleolus, and fixation of the PMF may therefore also reduce the need for syndesmotic screws.^14,18,21,34,49^ Several studies have demonstrated good clinical outcome and few complications using this posterior approach.^12,51^

Our clinic changed in 2015 toward more use of a posterior approach, aiming to improve clinical outcomes and patient satisfaction. Few studies have reported on the comparative outcomes after use of the traditional approach and the posterior approach for PMF fixation. The purpose of this study was therefore to compare the short-term patient-reported outcome measures (PROMs) and rate of complications in patients with ankle fractures including a PMF that were treated surgically with or without a posterior approach.

## Methods

All patients treated for ankle fractures with a low-energy mechanism of injury involving a PMF at a level 1 trauma hospital in Bergen, Norway, were eligible for inclusion in the study. A selective search through the operation planning system, Orbit version 5.11.2, was conducted based on Nordic Medico-Statistical Committee (NOMESCO) Classification of Surgical Procedures (NCSP) codes for bi- and trimalleolar fractures from January 2014 through December 2016. Radiographs from the time of injury were thereafter examined, so that only patients with an ankle fracture that involved the posterior malleolus were included. Included patients were invited to a follow-up evaluation involving questionnaires, clinical examination, and radiographs.

Exclusion criteria were deceased patients, follow-up at other hospital or in another country, high-energy mechanism, open fractures, former injury of the ipsilateral lower extremity causing current symptoms, and noncompliant patients. Patients with dementia and severe drug or alcohol abuse were considered noncompliant.

Patients were placed in groups according to the treatment approach given: group A (patients operated upon with a posterior approach) or group B (patients who received the traditional approach). To reduce bias in terms of differences in PMF size while analyzing outcomes across group A and group B, a one-to-one matching according to the size of the posterior malleolus fragment was performed. A size difference of maximum ±2% was allowed for within each matched pair.

**Figure 1.**
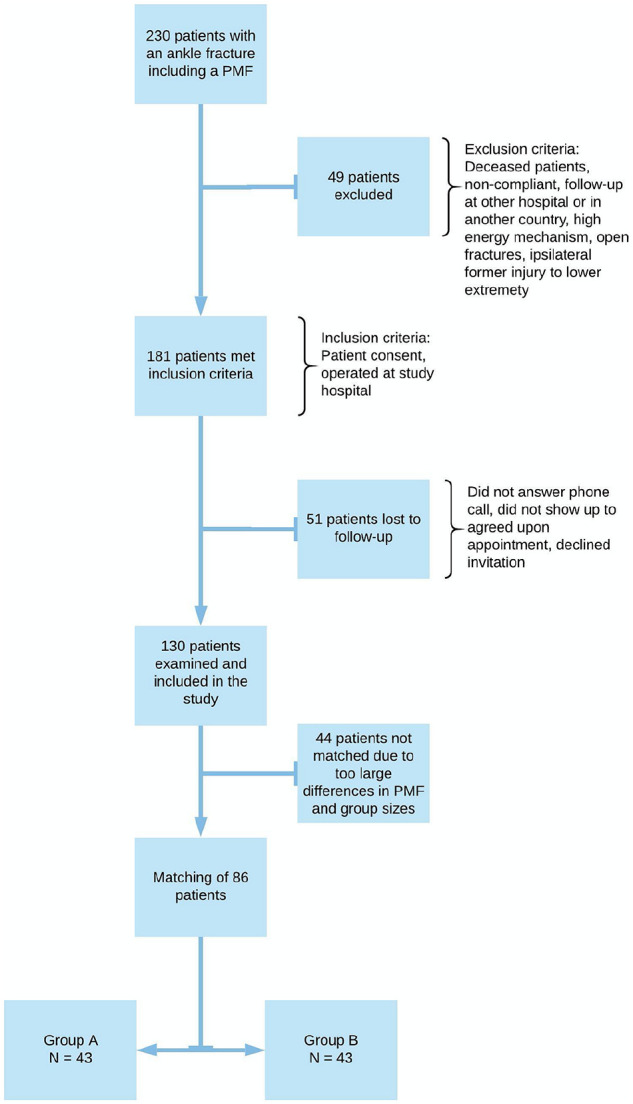
Search results, exclusion criteria, and inclusion criteria. In total, 130 patients met for a follow-up visit. To compare patients who received the traditional treatment (group B, n = 76 patients) to those operated through a posterior approach (group A, n = 54 patients), we matched patients one by one from each group according to the size of the posterior malleolus fragment. This rendered 86 patients, 43 in each group, for analysis. Due to too large discrepancies in the size of the posterior malleolus fragments (PMFs), 11 patients from group A and 33 patients from group B could not be matched.

Postoperative radiographs were assessed for intra-articular step-off after surgery. Patient selection and inclusion and exclusion criteria are illustrated in Figure 1.

In total, 130 patients were evaluated at a median 25 (interquartile range [IQR], 19-35 months) months after surgery. Median age was 57 (IQR, 41-67) years, 94 patients were female and 36 were male patients, and 79 fractures were classified as Weber B and 51 as Weber C. Median PMF size was 17% (IQR, 10%-26%). Median time from injury to operation was 5 (IQR, 0-9) days, median length of stay was 7 (IQR, 3-13) days, and median duration of surgery was 91 (IQR, 71-122) minutes.

### Surgical Technique

Fracture fixation was performed after standard principles of the Arbeitsgemeinschaft für Osteosynthesefragen (AO). In group A, patients were operated upon in a prone position. A posterolateral and, if needed, posteromedial direct approach was used. Ankle joint debridement was performed before the PMF was anatomically reduced. Fixation was achieved with 3.5-mm screws with or without a one-third tubular plate. The fibular fracture was reduced and fixed through the posterolateral incision while any medial malleolus fracture was addressed via a separate direct medial approach. Fibular plates were applied posteriorly on the fibula. The posterior approach was used when the PMF was planned to be fixed.

Patients in group B were treated in a supine position. The lateral and, if present, the medial malleolus fracture were treated first, through a direct lateral and direct medial approach. If the size of the PMF was considered 25% or more of the distal tibial articular surface on the lateral radiograph, the posterior malleolus fragment was thereafter fixed with anteroposterior, partially threaded, 3.5-mm cancellous screws. All posterior fragments had attempted indirect reduction by ligamentotaxis regardless of whether they were fixed or not.

In both groups, plating of the fibula fracture was performed with standard one-third tubular plates, standard plates, or anatomical locking compression plates (LCPs) depending on fracture type, bone quality, and comminution of the fracture.

In both groups, the ankle syndesmosis was tested for stability after fixation of the fractures with the Cotton test or external rotation at the surgeon’s discretion.^50^ If instability was seen, syndesmosis fixation was done with 1 quadricortical screw, 2 tricortical 3.5-mm screws, or a suture button.

Mobilization with partial weightbearing supported by crutches was allowed for the first 6 weeks. In cases of syndesmosis fixation, patients were allowed foot touch weightbearing for the first 6 weeks and thereafter partial weightbearing the next 6 weeks. Full weightbearing was allowed from 12 weeks in the latter cases. At our department, syndesmotic screws were routinely removed at 12 weeks with a planned operation at the outpatient clinic.

### Outcome Assessment

The primary outcome was Self-Reported Foot and Ankle Score (SEFAS).^5-8,15^ SEFAS was translated to Norwegian, and the translation was approved by the Center on Patient-Reported Outcome Data in Helse Bergen before use in patient evaluation. Median normative values of SEFAS are 48 for men and 47 for women, and the minimal important clinical difference has been reported to be a change of 5 points.^6,8^ As a generic quality-of-life assessment tool, we used the RAND-36,^17^ recently translated and validated into Norwegian by the Norwegian Institute of Public Health.^38^

Patients also completed a visual analog scale (VAS) of pain and VAS of satisfaction (0 meaning no pain/very unsatisfied and 10 meaning worst possible pain/very satisfied) to grade their level of pain and their level of satisfaction with surgery. VAS is a quick and easy way of assessing function that has commonly been used to evaluate outcomes after orthopedic surgery.^45^

PROMs in the matched patients were compared. Subanalyses were performed on patients with fragments smaller than 25%, comparing those who had the PMF fixed in group A to the patients in group B who did not have the PMF fixed. Also, the results of matched patients with the PMF fixed were compared.

Clinical examination included range of motion (ROM) in passive dorsi- and active plantarflexion and heel raise distance for both the operated and the uninjured ankle. Any differences between the sides were noted. Positive numbers denote larger movement of the uninjured ankle and negative numbers larger movement of the injured ankle. Dorsiflexion was performed with the foot being measured on top of a 2-step stool. The patient leaned forward as far as possible before the heel left the surface. The angle between the stool’s top surface and the anatomical axis of the fibula was measured with a goniometer. Plantarflexion was measured with the patient sitting on an examination bench with straight knees and actively plantarflexing the foot. The angle between neutral position and the axis of the fifth metatarsal was measured with a goniometer. Heel raise test was performed with the patients standing on a stool with one foot at the time. They would then perform a 1-leg heel raise. The distance between neutral and maximum height after heel raise was measured in centimeters.

Patient charts were reviewed for demographic data and information on fracture characteristics, time from injury to definitive operation, duration of operation, and length of stay. Complications were registered as surgical site infections, noninfectious skin problems, nerve injury, reoperations, mechanical irritation from the implant, and implant removal. Reoperation was defined as any new surgery due to malreduction of the fracture(s) or fixation of the syndesmosis after the primary operation.

**Figure 2.**
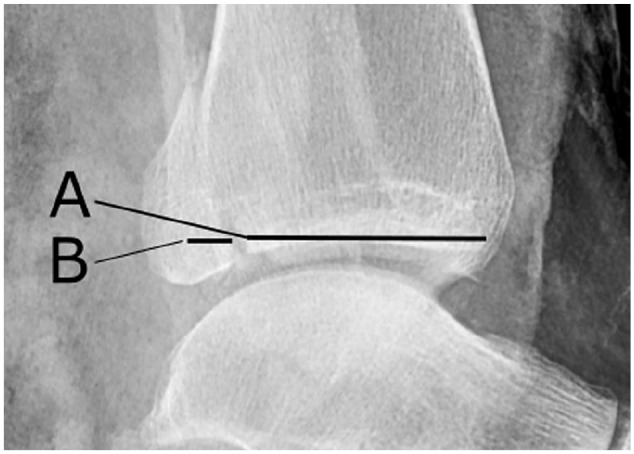
The size of the posterior malleolus fracture was measured as percentage joint involvement (B) of the anteroposterior length of the distal tibial articular surface (A + B) on lateral radiographs of the ankles ((B/(A + B)) *100 = % size of the distal tibial articulate surface).

**Figure 3.**
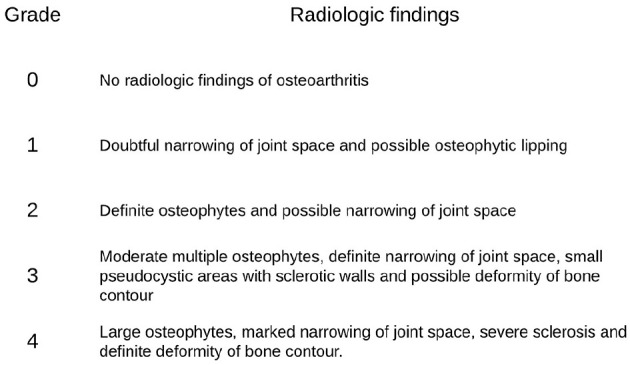
The Kellgren-Lawrence classification of grading of osteoarthritis.

The Weber classification and the Lauge Hansen classification were used to describe the fracture.^26^ The size of the PMF was measured as percentage of joint involvement of the anteroposterior length of the distal tibial articular surface on lateral radiographs of the ankle (Figure 2).^1^ Radiographs acquired at follow-up were examined by 2 of the authors, both experienced ankle surgeons. Grading of osteoarthritis (OA) was performed using the Kellgren and Lawrence classification (Figure 3).^24^

### Statistical Methods

Categorical variables were analyzed with Pearson χ^2^ test and nonparametric continuous variables were analyzed by Mann-Whitney *U* test. An a priori *P* value of <.05 was set to denote statistically significance. IBM SPSS version 24 (SPSS, Inc) was used for data management and analyses.

### Ethics

The Helse Bergen Data Protection Officer and Regional Committee for Medical and Health Research Ethics (REK) approved the project, REC ref.nr: 2016/1720. Informed consent was obtained from all patients before inclusion in the study.

## Results

At the follow-up evaluation, median SEFAS was 39 (IQR, 31-44) points, median RAND-36 was 78 (IQR, 59-88) points, median VAS of pain was 1 (IQR, 0-3), and median reported VAS of satisfaction was 8.5 (IQR, 7-10).

The matching procedure rendered 86 patients, 43 in each group, for analysis. Matching was not possible in 11 patients from group A and 33 from group B. When comparing patients included in the matching (n = 86) and those not included (n = 44), similar results were found between those groups in age, sex distribution, American Society of Anesthesiology class, severity of fracture, time from injury to operation, length of stay, use of temporary external fixator, infections, or other complications (all *P* > .1) Furthermore, there were no differences between groups in SEFAS (*P* = .53), RAND-36 (*P* = .39), VAS of pain (*P* = .23), or VAS of satisfaction (*P* = .91) at the follow-up evaluation. Also, similar results were found between patients in group A (n = 11) and group B (n = 33) within the unmatched patients.

### Comparison of Results in the Matched Patient Groups

No differences in patient demographics or fracture characteristics were found between matched patients across the groups (*ns*), but median time to follow-up was shorter (*P* < .01) in group A than in group B: 19 (range, 12-43) months vs 34 (range, 15-46) months (Table 1).

**Table 1.** Patient and Fracture Characteristics, Treatment Factors, and Complications.^a^

Characteristic	Group A (n = 43)	Group B (n = 43)	*P* value^b^
Demographics			
Female	28 (65)	35 (81)	.1
Male	15 (35)	8 (19)	
Age, median (IQR), y	53 (35-67)	60 (41-69)	.2
ASA ≥3	3 (7)	2 (5)	.6
Diabetes	2 (5)	1 (2)	.6
Smoking	4 (9)	5 (12)	.7
Fracture characteristics			
Weber class B/C	27 (63)/16 (37)	28 (67)/14 (33)^c^	.7
Lauge Hansen SER/PER	27 (63)/16 (37)	28 (65)/15 (35)	.09
Ankle fracture-dislocation	21 (49)	19 (44)	.7
PMF size,^d^ median (IQR), %	17 (12-24)	17 (12-24)	.99
Treatment summary			
Time from injury to definitive operation, median (IQR), d	8 (6-11)	0 (0-2)	<.001
Length of stay, median (IQR), d	12 (9-16)	3 (2-4)	<.001
Duration of operation, median (IQR), min	109 (89-147)	80 (60-103)	<.001
Fixation of PMF	42 (98)^e^	7 (16)	<.01
External fixator prior to operation	24 (56)	5 (12)	<.01
Syndesmotic fixation	6 (14)	21 (49)	<.01
Complications			
Infection	6 (14)	5 (12)	.8
Skin problems	12 (28)	2 (5)	<.01
Nerve injury	9 (21)	7 (16)	.6
Reoperations	3 (7)	3 (7)	1
Mechanical irritation	9 (21)	21 (49)	.01
Implant removal	3 (7)	27 (63)	<.01
Osteoarthritis grades 2-4	9 (21)	3 (7)	.06

Abbreviations: ASA, American Society of Anesthesiology; IQR, interquartile range; PER, pronation, external rotation; PMF, posterior malleolus fragment; SER, supination, external rotation.

a Values are presented as number (%) unless otherwise indicated. Group A: Patients operated upon in a prone position with a posterior approach to the ankle. Group B: Patients operated upon in a supine position with fixation of the PMF if the fragment was considered larger than 25% of the tibial articular surface, while smaller fragments were left unfixed.

b *P* values derived from Mann-Whitney *U* test for nonparametric continuous variables and Pearson’s χ^2^ test for categorical values.

c One patient in the traditional group did not have a fibular fracture; percentages calculated out of 42 patients.

d Measured as the percentage of the size of the PMF articular surface to the articular size of the distal tibia on a lateral radiograph.

e One patient got the posterolateral approach, but the PMF was not fixated as the surgeon considered the fragment to be well reduced.

Definitive surgery was performed within the first 24 hours of the injury in 30 (70%) patients in group B compared to 3 patients (7%) in group A (P < .001). At surgery, syndesmotic fixation was performed in 7 patients with Weber B and 20 patients with Weber C fractures, as well as in 5 of 8 patients with anteroposterior screw fixation of the PMF. In most patients, the quality of reduction of the PMF could not be assessed as the implants concealed the potential postoperative intra-articular step-off in the distal tibia on plain radiographs.

#### Outcomes at Follow-up Evaluation

**Figure 4.**
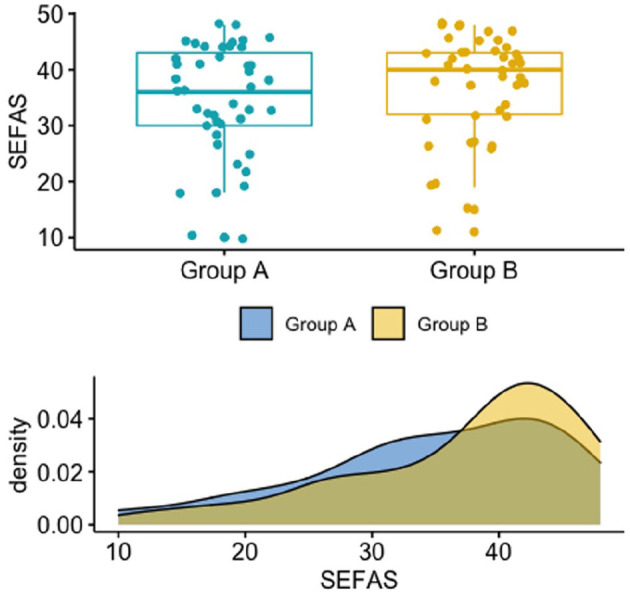
Boxplot (upper half) and density plot (lower half) showing the distribution of the Self-Reported Foot and Ankle Score (SEFAS, score from 0-48) in the 2 groups. n = 43 patients in each group. Each point in the boxplot graph represents a patient. The points are scattered for better visualization of the variation among the patients.

No differences were found between groups A and B in SEFAS, RAND-36, VAS of pain, and VAS of satisfaction (all *P* > .05) at the follow-up evaluation (Figure 4 and Table 2).

**Table 2.** Patient-Reported Outcome Measures at Follow-up of Matched Patients.^a^

Characteristic	Group A (n = 43), median (IQR)	Group B (n = 43), median (IQR)	*P* value^b^
PROM			
SEFAS	36 (30-44)	40 (32-43)	.2
RAND-36^c^	73 (54-88)	81 (55-89)	.6
VAS of pain^d^	2 (1-4)	1 (0-3)	.2
VAS of satisfaction^e^	9 (7-10)	8 (7-10)	.9

Abbreviations: IQR, interquartile range; PROM, patient-reported outcome measure; SEFAS, Self-Reported Foot and Ankle Score; VAS, visual analog scale.

a Group A: Patients operated upon in a prone position with a posterior approach to the ankle. Group B: Patients operated upon in a supine position with fixation of the posterior malleolus fragment if the fragment was considered larger than 25% of the tibial articular surface, while smaller fragments were left unfixed.

b *P* values derived from nonparametric continuous variables analyzed by Mann-Whitney *U* test.

c RAND-36: generic PROM for quality of life.

d 0 = no pain and 10 = worst possible pain. Pain score experienced the past 2 weeks prior to the clinical examination.

e 0 = very disappointed and 10 = very satisfied with the result.

The median difference in dorsiflexion, plantarflexion, and heel raise between the injured and noninjured ankle for group A was 10 (range, –1 to 27) degrees, 6.5 (range, –9 to 35) degrees, and 1.5 (range, –2 to 8) cm, respectively. Median differences in group B were 9 (range, –8 to 27) degrees, 5 (range, –50 to 35) degrees, and 1 (range, –6 to 8) cm, respectively. There were no statistically significant differences between the groups (all *P* > .05) (Table 3).

**Table 3.** Range of Motion.^a^

Characteristic	Group A (n = 43), median (IQR)	Group B (n = 43), median (IQR)	*P* value^b^
Difference in dorsiflexion	10 (5-19)	9 (4-15)	.3
Difference in plantarflexion	6.5 (2-12)	5 (0-10)	.2
Difference in heel raise (cm)	1.5 (0-3)	1 (0-3)	.2

Abbreviation: IQR, interquartile range.

a The difference in dorsiflexion and plantarflexion is measured in degrees on a goniometer. Positive numbers denote larger movement of the uninjured ankle and negative numbers larger movement of the injured ankle. Group A: Patients operated upon in a prone position with a posterior approach to the ankle. Group B: Patients operated upon in a supine position with fixation of the posterior malleolus fragment if the fragment was considered larger than 25% of the tibial articular surface, while smaller fragments were left unfixed.

b *P* values derived from Mann-Whitney *U* test for nonparametric continuous variables.

Subanalyses of patients with PMFs smaller than 25% comparing those who had fixation of the fragment in group A to the patients in group B who did not have fixation of the PMF revealed similar results between the groups (Table 4). Similar PROM results were also found among patients who got the PMF fixed (Table 5). The median PMF size among patients who had the PMF fixed was 34% (IQR, 26%-39%) in group A and 35% (IQR, 26%-39%) in group B (*P* = .6). The median time to follow-up was 31 (IQR, 19-41) months in group A and 35 (IQR, 34-40) months in group B (*P* = .6)

**Table 4.** Subanalyses of Patients With Posterior Malleolus Fragment Size Smaller Than 25%.^a^

Characteristic	Group A: PMF fixed (n = 31), median (IQR)	Group B: PMF not fixed (n = 34), median (IQR)	*P* value^b^
PROM			
SEFAS	36 (27-42)	40 (27-43)	.2
RAND-36^c^	68 (57-88)	76 (46-88)	.8
VAS of pain^d^	2 (1-5)	1.5 (0-4)	.2
VAS of satisfaction^e^	8 (6-10)	8 (7-10)	.9

Abbreviations: IQR, interquartile range; PMF, posterior malleolus fragment; PROM, patient-reported outcome measure; SEFAS, Self-Reported Foot and Ankle Score; VAS, visual analog scale.

a Patient-reported outcome measures at follow-up by surgical approach in patients with fragments smaller than 25%, comparing those who had the PMF fixed in the posterior approach group to the patients in the traditional approach group who did not have the PMF fixed. Group A: Patients operated upon in a prone position with a posterior approach to the ankle. Group B: Patients operated upon in a supine position with fixation of the posterior malleolus fragment if the fragment was considered larger than 25% of the tibial articular surface, while smaller fragments were left unfixed.

b *P* values derived from Mann-Whitney *U* test for nonparametric continuous variables.

c RAND-36: generic PROM for quality of life.

d 0 = no pain and 10 = worst possible pain. Pain score experienced the past 2 weeks prior to the clinical examination.

e 0 = very disappointed and 10 = very satisfied with the result.

**Table 5.** Subanalyses of Matched Patients With Fixed Posterior Malleolus Fragment (PMF).^a^

Characteristic	Group A (n = 7), median (IQR)	Group B (n = 7), median (IQR)	*P* value^b^
PROM			
SEFAS	41 (30-44)	43 (38-45)	.3
RAND-36^c^	87 (73-88)	90 (85-92)	.3
VAS of pain^d^	1 (1-3)	1 (0-2)	1
VAS of satisfaction^e^	10 (9-10)	10 (9.5-10)	.6

Abbreviations: IQR, interquartile range; PROM, patient-reported outcome measure; SEFAS, Self-Reported Foot and Ankle Score; VAS, visual analog scale.

a Patient-reported outcome measures at follow-up, with comparison of matched patients from groups A and B with the PMF fixed. Group A: Patients operated upon in a prone position with a posterior approach to the ankle. Group B: Patients operated upon in a supine position with fixation of the PMF if the fragment was considered larger than 25% of the tibial articular surface, while smaller fragments were left unfixed.

b *P* values derived from Mann-Whitney *U* test for nonparametric continuous variables.

c RAND-36: generic PROM for quality of life.

d 0 = no pain and 10 = worst possible pain. Pain score experienced the past 2 weeks prior to the clinical examination.

e 0 = very disappointed and 10 = very satisfied with the result.

#### Complications

Overall, 7% (6 of 86) patients were treated for a deep infection in the operated ankle—2 of 43 (5%) in group A and 4 of 43 (9%) in group B (*ns*). Mechanical irritation was reported by 17 patients in group B and 6 patients in group A located at 1 or both of the lateral and medial malleoli (*ns*). In group B, 15 patients had planned, routine implant removal. Ten patients removed implants due to mechanical irritation, and 2 further patients removed implants due to an infection. In group A, mechanical irritation led to implant removal in 3 cases (Table 1).

Radiographs taken at follow-up revealed more patients with higher grade of osteoarthritis in group A (*P* = .06) (Table 1).

## Discussion

Patient-reported outcomes were similar in patients who had their ankle fracture, involving a posterior malleolus fragment, treated compared to those who did not. Patients in the latter group more frequently received temporary external fixation prior to definitive surgery, waited longer for definitive surgery, had longer length of stay, had more postoperative noninfectious skin problems, and displayed more cases of severe posttraumatic osteoarthritis. Those treated in group A, however, experienced less mechanical irritation, less frequently had implant removal, and less frequently required additional syndesmotic stabilization. Only 7 of the 43 patients in group B had fixation of the PMF. Rate of infection, nerve injury, and reoperation were similar between the groups.

The difference between the 2 groups in length of stay and time from injury to surgery could be explained by the practice at our department in the study period. From the autumn of 2015, an increasing number of patients were treated using a posterior approach, but only a few surgeons were familiar with this method. Consequently, some patients were primarily treated with an external fixator in the absence of the appropriate surgeon. The aim of delaying surgery was to achieve better postoperative results, and the patients were kept in-house until definitive surgery, which was further postponed by waiting for the soft tissue swelling to resolve. External fixation was chosen for better control of the ankle fracture and to facilitate better inspection of the skin and soft tissue swelling. We also wanted to avoid potential dislocation of the ankle every time the plaster cast would be opened for inspection of the swelling. However, the results of this study show no improvement of this treatment strategy. As the fracture characteristics display, there were no differences in mechanism of injury or fracture classification. We would therefore argue that the increased time from injury to surgery, longer length of stay, and more frequent soft tissue challenges in group A reflect this practice rather than more severe injuries in this group of patients. Despite the differences in time to surgery and noninfectious skin problems in our study, no difference in clinical and patient-reported outcome was found between the groups.

Compared to the normative values of SEFAS,^8^ our results of median 36 for group A and 40 for group B reflect the serious impact on function and quality of life of an ankle fracture involving the posterior malleolus. Mason et al^29^ also reported low PROM scores in patients with posterior malleolus fractures, with a mean Olerud-Molander Ankle Score of 74.1. Xu et al^56^ found an average American Orthopaedic Foot & Ankle Society (AOFAS) score of 95.9 in a similar population. Xu et al^56^ could not find any difference in treatment effect between fixation and nonfixation of the PMF. Both groups in the current study reported similar RAND-36, VAS of satisfaction, and VAS of pain like De Vries et al^10^ and Langenhuijsen.^25^ Loss of dorsiflexion is known as a predictor of outcome after ankle fractures^16^; we did not find any differences in range of motion between the 2 groups in the current study.

The size of the PMF and the need for fixation is a matter of ongoing debate. Some authors report no difference in outcome in patients with fixation and without fixation of smaller fragments, and they more conventionally recommend fixation if the PMF involves 25% or more of the articular surface.^9,10,33,35,44,55^ Other authors recommend ORIF of all PMFs regardless of their size as this this was found to reduce the need for syndesmotic fixation and improve outcomes in their study.^3,23,29,30^ The subanalyses of patients with PMF smaller than 25% in the current study displayed similar SEFAS scores between treatment groups, although fragments were fixed in group A and no fixation was performed in group B. There was a trend of better results in group B. Also, PROM results were similar when comparing patients who had their PMF fixed across treatment groups. These patients also had similar time to follow-up. However, comparison was difficult due to the small number of patients. Both subanalyses suggest that the treatment in group B gave equally good results as the posterior approach, used in group A. Some authors suggest that clinical outcome is related to fracture displacement, articular surface congruency, and residual tibiotalar subluxation, rather than PMF size.^39,45,48^ Several studies,^11,53,56^ including a review from 2018 by Verhage et al,^52^ argue that postoperative step-off is the most important factor predicting posttraumatic osteoarthritis. The current study showed a surprising trend toward more osteoarthritis in group A (*P* = .06). The result is surprising as we expected less osteoarthritis and pain in this group of patients due to shorter time to follow-up and proposed better fracture reduction. One could speculate whether fractures in group A were more comminuted than seen on lateral radiographs and that the degree of soft tissue injuries was worse than those in group B. Additional computed tomography (CT) scans would have given more detailed information on preoperative severity of the fracture—and postoperative reduction—but were not available for this patient cohort. In most patients, the quality of reduction of the PMF could not be assessed as the implants concealed the potential postoperative intra-articular step-off in the distal tibia on plain radiographs.

The current finding of a lower rate of syndesmotic stabilization in group A, in whom a posterior approach was used, is also in accordance with other studies.^14,27,34,49^ However, the use of the posterior approach could serve as bias toward not fixating the syndesmosis even if it was slightly unstable. One could speculate whether this could explain the present increased rate of high-grade osteoarthritis in the group of patients treated with this approach. These patients had less mechanical irritation and less frequently required implant removal. These findings are consistent with other reports and illustrates that the posterior approach gave better soft tissue coverage than when the direct lateral approach was applied for fixation of the fibular fracture.^28,42^ The postoperative protocols could also serve as bias. Nearly half of the patients in group B had syndesmotic fixation and were not allowed to bear full weight until after 3 months. The difference in follow-up time could also serve as a bias for the reported PROM and clinical outcomes. Patients from group B had a longer median follow-up time and could therefore have a higher degree of adaptation to the state of their previously injured ankle. Patients from group A, with the more recent injury fresh in mind, might have a lesser degree of adaptation and therefore report worse function than if follow-up time was equal between groups.

Furthermore, there was an evident difference in time from injury to definitive surgery, where most of the patients in group B were operated on within the first day of admission. The literature in general recommends definitive surgery as early as possible.^4,19,36,43,47^ Therefore, if use of the posterior approach leads to a delay in surgery, this adds to the discussion on the benefit of changing approaches.

The SEFAS questionnaire was chosen as the primary outcome as it is validated for patients with ankle fractures—and normative values from the general population have been established.^8^ Across several PROMs, SEFAS is considered to have the best measurement properties for the current population.^15^ Further strengths include use of a multitude of outcome measures, radiographs, and complication rates. This gives a more complete picture of the effectiveness of the different approaches for treating ankle fractures. The current study is a transparent evaluation of clinical practice and change in operative treatment at a level 1 trauma hospital. The use of one-to-one matching allowed for comparison of outcomes in similar fractures treated with 2 different approaches. The evident similarities in fracture characteristics and soft tissue injuries support this as a basis for comparison across a traditional and a more novel surgical approach.

The retrospective study design has several well-known limitations. In the current study, only 130 (72%) of the 181 eligible patients were available for the follow-up evaluation. The reasons for nonparticipation varied, but we cannot rule out a selection bias. The current exclusion criteria were chosen as high-energy injuries and open fractures have a different soft tissue prognosis than fractures with lower energy. Furthermore, although a matching algorithm was applied, to adjust for potential differences that could bias the outcome, patients likely hold a certain degree of heterogeneity. As this report is on the first patients operated upon with a new technique, the results might also reflect a certain learning curve. The results with the posterior approach could therefore improve with time—displaying the need for an ongoing evaluation of results after surgery. The more frequent use of temporary stabilization prior to definitive surgery in group A could have led to a prolonged length of stay and more noninfectious skin complications. If all patients had undergone definitive surgery within 24 hours, this potential effect on outcomes would have been eliminated. Finally, several studies have shown that pre- and postoperative CT scans are preferred over radiographs to accurately assess the anatomy of the PMF and the quality of fracture reduction.^32-34^ Unfortunately, only radiographs were available in the current patient series.

### Conclusion

In the current study, clinical outcomes of patients treated for ankle fractures involving PMFs were not improved by reduction and fixation, through a posterior approach, compared to a traditional indirect reposition and anteroposterior fixation. Most of the patients in the traditional group did not have fixation of the PMF. Among patients with a PMF smaller than 25%, patients in the group without fixation reported similar results to those who got fixation in the posterior approach group. Also, matched patients with the PMF fixed from each group reported similar results. Although the need for syndesmotic fixation was reduced with the change to a posterior approach, patients waited longer until definitive surgery, had longer length of stay, more frequently developed severe posttraumatic osteoarthritis, and more frequently reported noninfectious skin problems. Although limitations apply, these results challenge the view that all posterior malleolus fractures need fixation.

## Supplemental Material

FAI969431_disclosures – Supplemental material for Traditional Approach vs Posterior Approach for Ankle Fractures Involving the Posterior Malleolus Click here for additional data file. Supplemental material, FAI969431_disclosures for Traditional Approach vs Posterior Approach for Ankle Fractures Involving the Posterior Malleolus by Kristian Pilskog, Teresa Brnic Gote, Heid Elin Johannessen Odland, Knut Andreas Fjeldsgaard, Håvard Dale, Eivind Inderhaug and Jonas Meling Fevang in Foot & Ankle International
